# Brain region-specific expression of genes mapped within quantitative trait loci for behavioral responsiveness to acute stress in Fisher 344 and Wistar Kyoto male rats

**DOI:** 10.1371/journal.pone.0194293

**Published:** 2018-03-12

**Authors:** Jeanie K. Meckes, Patrick H. Lim, Stephanie L. Wert, Wendy Luo, Stephanie A. Gacek, Dana Platt, Ryan Jankord, Kathrin Saar, Eva E. Redei

**Affiliations:** 1 Department of Psychiatry and Behavioral Sciences, Feinberg School of Medicine, Northwestern University, Chicago, IL, United States of America; 2 Applied Neuroscience, Warfighter Interface Division, 711th Human Performance Wing, Air Force Research Laboratory, Wright-Patterson Air Force Base, OH, United States of America; 3 Max Delbrück Center for Molecular Medicine in the Helmholtz Association, Berlin-Buch, Germany; Technion Israel Institute of Technology, ISRAEL

## Abstract

Acute stress responsiveness is a quantitative trait that varies in severity from one individual to another; however, the genetic component underlying the individual variation is largely unknown. Fischer 344 (F344) and Wistar Kyoto (WKY) rat strains show large differences in behavioral responsiveness to acute stress, such as freezing behavior in response to footshock during the conditioning phase of contextual fear conditioning (CFC). Quantitative trait loci (QTL) have been identified for behavioral responsiveness to acute stress in the defensive burying (DB) and open field test (OFT) from a reciprocal F_2_ cross of F344 and WKY rat strains. These included a significant QTL on chromosome 6 (*Stresp10*). Here, we hypothesized that the *Stresp10* region harbors genes with sequence variation(s) that contribute to differences in multiple behavioral response phenotypes between the F344 and WKY rat strains. To test this hypothesis, first we identified differentially expressed genes within the *Stresp10* QTL in the hippocampus, amygdala, and frontal cortex of F344 and WKY male rats using genome-wide microarray analyses. Genes with both expression differences and non-synonymous sequence variations in their coding regions were considered candidate quantitative trait genes (QTGs). As a proof-of-concept, the F344.WKY-*Stresp10* congenic strain was generated with the *Stresp10* WKY donor region into the F344 recipient strain. This congenic strain showed behavioral phenotypes similar to those of WKYs. Expression patterns of *Gpatch11* (G-patch domain containing 11), *Cdkl4* (Cyclin dependent kinase like 4), and *Drc1* (Dynein regulatory complex subunit 1) paralleled that of WKY in the F344.WKY-*Stresp10* strain matching the behavioral profiles of WKY as opposed to F344 parental strains. We propose that these genes are candidate QTGs for behavioral responsiveness to acute stress.

## Introduction

Acute stress is a common occurrence in everybody’s life with differing severity. Individual variation in the response to acute stress depends on the sensitivity to the stressor and the ability to cope with it. Genetic studies support the assumption that acute stress responsiveness is a quantitative trait [1–3]. Its narrow-sense heritability ranges between 0.4–0.6 defined by multivariate genetic analyses in family and twin studies [4–7]. The heritability of acute stress responsiveness is estimated to be similar in rodents to that in humans [8, 9]. However, only a limited number of candidate genes have been proposed for individual variation in behavioral acute stress responsiveness to date [10–14]. The purpose of this study was to identify candidate genes with sequence variations that contribute to variations in individual stress vulnerability.

We have studied the genetic components of behavioral responsiveness to acute stress in the reciprocally crossed F_2_ generation of Wistar Kyoto (WKY) and Fischer 344 (F344) rat strains using the defensive burying (DB) and open field tests (OFT) [15–19]. The parental WKY strain consistently exhibits hypoactive and avoidant behavior compared to that of the F344 [19]. We argue that this consistent repertoire of behavior across various tests is not composed of discrete, individual reactions to each situation, but belies a more global response to an acute challenge with substantial genetic origin. Among the quantitative trait loci (QTL) found in these studies, a highly significant locus on chromosome 6, *Stresp10*, has been identified for multiple phenotypes; a potential pleiotropy for behavioral stress responsiveness (Table 1) [16]. The phenotypes associated with this QTL include latency to bury, grooming, rearing in DB, and rearing in OFT. It is notable that a discrepancy was described for the OFT and DB rearing QTLs using genetic mapping from the time of the original publication [19]. However, physical mapping using the more recent rat genome version Rnor_6.0 identified that these QTLs indeed overlap. Furthermore, rearing measures highly correlate between the two tests [19]. All of these behaviors represent a general stress response to an acute stressor, whether it be the shock in the DB or the novelty of OFT. Thus, this chromosomal region is likely to harbor one or more genes with sequence variation(s) that contribute to phenotypic variations in general stress responsiveness to acute stressors between these strains.

**Table 1:** List of behavioral phenotypes associated with the *Stresp10* QTL.

QTL Symbol	Position	Behavior	LOD	Pointwise significance	Variance (%)	Reference
*Stresp10*	6:1–35,623,029	Latency (DB)	3.55	0.00002	3.5	[15]
		Rearing (DB)*^	3.72	0.00019	1.6	[19]
		Grooming (DB)*	3.1	0.00079		[19]
		Rearing (OFT)	8.24	2.82E-10	8.9	[19]

* suggestive, trait x lineage covariates

^ Rearing (DB) QTL was originally identified at 62cM

From the Rat Genome Database version Rnor_6.0

In this study, we aimed to identify candidate quantitative trait genes (QTG) that contribute to differences in acute stress responsiveness between F344 and WKY rat strains in the *Stresp10* QTL. To aid in the identification of QTG(s), first we established the differentially expressed genes (DEGs) between the two parental strains within the *Stresp10* QTL using genome-wide microarray analyses in the hippocampus, amygdala, and frontal cortex. Using the Rat Genome Database (www.rgd.mcw.edu) and the original sequences of the two parental strains [12, 14], we then determined nonsynonymous sequence variations in the coding regions of DEGs between the F344 and WKY strains. We hypothesized that DEGs that show expression differences parallel to behavioral differences between the two strains are candidate quantitative trait genes. To investigate the generalizability of the findings to a different acute stress phenotype, we measured the behavioral response differences in the conditioning part of the contextual fear conditioning (CFC) paradigm between the two strains since the acute stress of the electric shock in the CFC is similar to that shock in the DB test. For proof-of-concept, we measured the transcript levels of these candidate QTGs in the brain regions of a congenic strain generated by inserting the *Stresp10* WKY donor region into the F344 recipient strain. DEGs with expression profiles in the F344.WKY-*Stresp10* congenic strain similar to the WKY’s, but different from the F344, suggest that the sequence variations within these genes might contribute to the *Stresp10* QTL.

## Materials and methods

### Animal care and treatment

All animal experiments were performed according to procedures approved by the Institutional Animal Care and Use Committee of Northwestern University. Adult male Fischer 344 (F344) and Wistar Kyoto (WKY) rats (approximately 3 months old) were obtained from Envigo (Indianapolis, IN) and Charles River Laboratories (Wilmington, MA), respectively. All animals were group-housed (2–3 per cage) in a temperature-controlled environment with 12 h light/dark cycles and allowed feed and water *ad–libitum*.

The experimental design required three animal cohorts. Specifically, one cohort of control F344 and WKY rats were not subjected to any behavioral testing. RNA was isolated from the three brain regions and used for the microarray experiments. The second cohort consisted of F344 and WKY rats that were subjected to CFC testing and their RNA was isolated from the three brain regions. This RNA was used for expression analyses by RT-PCR of candidate genes together with RNA isolated from the F344.WKY-*Stresp10* rats. The third cohort of F344, WKY, and F344.WKY-*Stresp10* rats were maintained independently and used for only behavioral testing in the OFT followed by DB three weeks later.

### Construction of congenic F344.WKY-Stresp10

The F344.WKY-*Stresp10* congenic strain was generated by repeated marker-directed backcrossing of the F344-WKY F_1_ generation into the F344 parental strain. The markers used are described before [16, 19]. The N_15_ generation of F344.WKY-*Stresp10* congenic male animals (approximately 3 months old) were used for behavioral experimentation together with the simultaneously maintained F344 and WKY rats. The 15^th^ generation of the F344.WKY-*Stresp10* strain was homozygous from 135,053 to 6,709,713 bp and from 19,464,437 to the end of the QTL at 28,931,796 bp, and heterozygous from 6,963,239 to 15,597,330 bp (S1 Table). Additionally, after 15 generations of backcross, congenic strains are known to have less then 5% heterozygosity throughout the genome [20]. This strain was characterized using the 10K Affymetrix Targeted Genotyping Array (Affymetrix, Santa Clara, CA) [21]. Using this array, 4 μg of rat DNA was genotyped with the GeneChip Scanner 2000 Targeted Genotyping System (GTGS) following the manufacturer’s protocol (Affymetrix, Santa Clara, CA) [22]. The genotyping data was analyzed using the corresponding software for GTGS (Affymetrix, Santa Clara, CA). The 10K Affymetrix Targeted Genotyping Array used the rat genome version RGSC_3.4. The QTL locations were translated to the latest version or the rat genome, Rnor_6.0. S1 Table lists the QTL locations from both versions of the rat genome.

### Behavioral tests

#### Contextual fear conditioning

The fear conditioning phase of the contextual fear conditioning test was carried out as described previously [23]. Briefly, male rats of the F344 and WKY strains were individually placed in the CFC chamber (Technical & Scientific Equipment, Bad Homburg, Germany) and exposed to 3 minutes of habituation followed by 3 footshocks (0.8 mA, 1 sec duration, spaced 1 minute apart). The behavior observed after the footshocks was analyzed by the TSE Videomot software version 5.75 (Bad Homburg, Germany), which measures the animals’ movement, including distance travelled, number of times rearing, and freezing duration. The rats were sacrificed by quick decapitation 24 hours later. Whole brains were collected in RNAlater (Ambion, Austin, TX) and frozen at -80°C until dissection of brain regions. Total RNA was isolated from the hippocampus, amygdala, and frontal cortex, and was used for the expression analyses of candidate genes. All rats were approximately 3 months old at the time of sacrifice and tissue sample collection.

#### Open field test

The open field test was carried out on another cohort of animals, as previously described [23]. Briefly, male rats of the F344, WKY, and F344.WKY-*Stresp10* strains were placed in a circular arena (82 cm in diameter) with a 30 cm high wall, lit to a brightness of approximately 60 lux by indirect overhead lighting. The arena contained an inner concentric circle (50 cm in diameter) designated as the inner zone. The rats were placed in the center of the arena and allowed to move freely for 10 min while the activity was recorded and tracked by the TSE Videomot software 2 version 5.75 (Bad Homburg, Germany), which measured the number of times rearing by the animal.

#### Defensive burying

Three weeks after the OFT, the defensive burying test was carried out on the same animals as previously described in the QTL studies [15, 16, 19]. Briefly, male rats of the three strains were habituated to a Plexiglas chamber (40 cm square, 60 cm high) with bedding (wood shaving) (7 cm deep, 1 cm below the hole for the prod) for 15 min each day, for three consecutive days, between 10:00 AM and 2:00 PM. On the fourth day, a continuously electrified prod was introduced into the chamber, which delivered a 4.5 mA shock when the rat touched it. The shock was generated from a shock generator (Lafayette Instruments, San Diego, CA) set at 4.5 mA. The rats typically explored the novel prod and received a shock, which started the 15-minute video-taped test. Once shocked, animals typically retreated to the back of the cage, and either remained there (classic WKY behavior) or began spraying bedding toward the prod in an effort to cover it. Behaviors scored by an observer blind to the genotype of the animal include the latency to begin burying, the total time spent burying (duration of burying), the duration of grooming, and the number of times rearing by the animal. The rats were sacrificed by quick decapitation two weeks after the test. Whole brains were collected in RNAlater (Ambion, Austin, TX) and frozen at -80°C until dissection. All rats were approximately 3 months old at the time of sacrifice and tissue sample collection.

### Brain dissection and RNA isolation

Brain regions (hippocampus, amygdala, and frontal cortex) were dissected from adult male F344, WKY and F344.WKY-*Stresp10* congenic rats as described previously [24] and stored in RNAlater (Ambion, Austin, TX) at -80°C. Briefly, a rat brain matrix was used to prepare primary sections from which the brain regions were dissected according the Paxinos coordinates [25]: hippocampus (AP−2.12 to −6.0, ML 0 to 5.0, DV 5.4 to 7.6), amygdala (AP −0.58 to −2.18, ML 1.5 to 4.5, DV 4 to 5.75), and frontal cortex (AP 5.20 to 1.70, ML 0 to 3.3, DV 9.0 to 4.4).

Total RNA was isolated from individual tissue samples using the Direct-zol RNA MiniPrep Kit (Zymo Research, Irvine, CA). Briefly, individual tissue samples were homogenized in TRIzol reagent (Ambion, Austin, TX) and RNA was immediately isolated using the kit following the manufacturer’s protocol. Total RNA (2 μg) was reverse transcribed to generate double-stranded cDNA using the SuperScript VILO cDNA Synthesis Kit (ThermoFisher Scientific, Waltham, MA).

### Microarray analysis

Genome-wide microarray analysis was performed from RNA isolated from hippocampi, amygdalae, and frontal cortexes of F344 and WKY adult male rats that were unstressed, without undergoing any behavioral testing, as described previously [26]. Briefly, the cDNA generated from the RNA was linearly amplified and labeled with biotinylated nucleotides in an *in vitro* transcription reaction using the Illumina TotalPrep RNA Amplification Kit (San Diego, CA) to make cRNA. 1.5 μg of biotin-labelled and fragmented cRNA was then hybridized onto RatRef-12 Expression BeadChips (Illumina, San Diego, CA). The BeadChips have multiple probes per transcript to mitigate hybridization bias artifacts. Probe intensity data from the BeadChips were directly read into the R software environment (http://www.R-project.org) from bead summary files produced by BeadStudio using the R/beadarray package [26, 27]. Quantile normalization was applied to the data using the R/preprocessCore package [26, 28]. Data quality was assessed using histograms of signal intensities, scatter plots, and hierarchical clustering of samples, as previously described [26]. Statistical significance of microarray expression differences between F344 and WKY was determined using ANOVA methods within the R/maanova package as previously described [26, 29]. DEGs were determined between strains with an FDR-adjusted *P*–value less than 0.05 and a fold change greater than 1.3 (30% increase or decrease). This criterion has been well-established to give biologically meaningful datasets when interpreting differential gene expression profiles in microarray experiments [30, 31]. The microarray data used the rat genome RGSC_v3.4 for identification of transcripts, which was translated to the latest version of rat genome, Rnor_6.0.

The differential gene expression profiles between the two strains were determined with a significance criteria of an *FDR*-adjusted *P* value less than 0.05 and an absolute fold change above 1.3 (30% increase or decrease). With these criteria, we found 1,030 DEGs in the hippocampus, 769 in the amygdala, and 976 in the frontal cortex, as listed in S3–S5 Tables.

### Identification of single nucleotide polymorphisms in coding regions

The genes with non-synonymous single nucleotide polymorphisms (SNPs) within coding regions between the F344 and WKY genomes were obtained from the Rat Genome Sequencing and Mapping Consortium and Baud et al. [12, 14]. In these studies, both F344 and WKY genomes were first mapped to the Brown Norway reference genome, version RGSC_3.4. Using the Integrative Genomics Viewer (Broad Institute, Cambridge, Massachusetts), we identified the coding sequence variations between the F344 and WKY genomes and translated them to the rat genome version Rnor_6.0. For quality control, we set the criteria that the coverage for each SNPs had to be greater than 10 reads, where reads are used to reconstruct the sequence. The more reads a sequence had, the more reliable the data. Furthermore, the single nucleotide variations had to be called in at least 50% of the reads.

Among inherited gene variations in humans, nonsynonymous single nucleotide polymorphisms that lead to an amino acid change in the protein product are most relevant to human inherited diseases [32]. Therefore, as the first step in the identification of candidate QTGs, we focused on this subset of genes. Genes with non-synonymous SNPs within the heterozygous *Stresp10* region were excluded from our analysis. Of the 16 DEGs mapped within the *Stresp10* region, 10 genes were found to contain SNPs (Table 2). SNPs are listed in S9 Table.

**Table 2:** Differentially expressed genes within the *Stresp10* QTL region containing coding region non-synonymous SNPs between F344 and WKY.

Gene Symbol	Gene Description	Chr	Start	End	*P-value* 0 *= <* 1.00E-08
Gpatch11^*h*,*a*,*f*^	G-patch domain containing 11	6	1410507	1423041	*h*: 0, *a*: 0, *f*: 0
Prkd3^*f*^	Protein kinase D3	6	1546018	1622232	*f*: 1.18E-07
Cyp1b1^*h*,*f*^	Cytochrome P450, family 1, subfamily b, polypeptide 1	6	2307808	2316722	*h*: 0, *f*: 0
Cdkl4^*h*^	Cyclin-dependent kinase-like 4	6	3234090	3254779	*h*: 7.39E-05
Mta3^*h*,*f*^	Metastasis associated 1 family, member 3	6	6908684	7031828	*h*: 0, *f*: 0
Ttc27^*a*,*f*^	Tetratricopeptide repeat domain 27	6	21735834	21880003	*a*: 1.42E-07, *f*: 0
Alk^*h*,*a*,*f*^	Anaplastic lymphoma receptor	6	22696397	23203775	*h*: 0, *a*: 0, *f*: 0
Rbks^*h*^	Ribokinase	6	26051396	26128906	*h*: 0
Drc1^*a*^	Dynein regulatory complex subunit 1	6	27425235	27460038	*a*: 4.74E-08
Dtnb^*h*,*a*,*f*^	Dystrobrevin, beta	6	27975417	28177214	*h*: 5.09E-07, *a*: 3.79E-05, *f*: 1.56E-05

*h*: hippocampus, *a*: amygdala, *f*: frontal cortex

From the Rat Genome Database version Rnor_6.0

### Real-time RT-PCR

The real-time reverse transcription-polymerase chain reaction (qRT-PCR) was carried out using RNA from the second cohort of F344 and WKY rats that were subjected to CFC testing, as well as RNA from the F344.WKY-*Stresp10* rats. Primers for each gene were designed using Primer Express Software version 3.0 (Applied Biosystems, Carlsbad, CA). The default setting was used to design primers that amplify 80 to 150 bp regions. The primer sequences for the candidate quantitative trait genes are listed in S2 Table. Five ng of cDNA were amplified in 20 μL reactions (1X SYBR Green Master Mix (ThermoFisher Scientific), 250 μM primers) in QuantStudio 7 Flex Real-Time PCR System (ThermoFisher Scientific, Waltham, MA) using the relative quantification (-ΔΔCt) method, with *Gapdh* as the housekeeping gene and a general calibrator. We have established that there was no change in hippocampal *Gapdh* expression across strains and conditions.

### Statistical analysis

All data were presented as mean ± standard error of mean. Outliers from the quantitative RT-PCR data were determined as being more than two standard deviation away from the mean. Therefore, the number of samples per group differ between target genes. All statistical analyses were performed using GraphPad Prism v 7.0 (GraphPad Software, La Jolla, CA). Statistical significance of differences between strains were determined by ANOVA, followed by post-hoc analysis with the Bonferroni’s correction for multiple comparisons. Statistical significance was considered at an adjusted *P*-value of less than 0.05. When significant main effects were indicated by the ANOVA, but the Bonferroni’s multiple comparisons test did not show significance, hypothesis testing by Student’s *t*-test was carried out between groups. Our decision to apply the Student’s *t-*test was based on an increasing number of discussions arguing that *P-*values are not as reliable as it is previously thought [33] and that while a three-group comparison ANOVA may not result in significance, two groups of the three can differ from each other at the *P* < 0.05 level [34]. ANOVA results are given in the results and post-hoc significances are noted in the figures.

## Results

### Microarray analysis

Genome-wide microarrays were performed on hippocampal, amygdalar, and frontal cortex RNA from unstressed F344 and WKY male rats. To identify potential QTGs, DEGs were mapped within the *Stresp10* QTL chromosomal region. This region is associated with multiple behavioral phenotypes in response to acute stress (Table 1). The chromosomal location of these QTGs was mapped from RGSC_v3.4 to the latest version of rat genome Rnor_6.0. Of the DEGs, 14 genes in the hippocampus, 12 in the amygdala, and 14 in the frontal cortex were found within the *Stresp10* region (S6–S8 Tables). Since many of these genes overlap in two or more brain regions, there were a total of 18 genes to investigate. Precisely, there were six genes (*Gpatch11*, *Slc3a1*, *Camkmt*, *Alk*, *Dtnb*, *Klhl29*) that overlapped in all three brain regions: *Rasgrp3* between the hippocampus and amygdala, *Cyp1b1* and *Mta3* between the hippocampus and frontal cortex, and *Ttc27* between the amygdala and frontal cortex.

### Single nucleotide polymorphisms in coding regions of DEGs mapped within *Stresp10*

To determine which genes contribute to the phenotypic variation in acute stress responsiveness, we identified the genes with non-synonymous single nucleotide polymorphisms (SNPs) within coding regions between the F344 and WKY genomes, which were obtained from Baud et al. [12, 14]. The SNPs were identified first between the WKY, F344 and the Brown Norway reference genome and then between the WKY and F344 genome using Rnor_6.0. The candidate SNPs had to be greater than 10 number of reads and the single nucleotide variations in greater than 50% of the reads. Of the 16 DEGs mapped within the homozygous *Stresp10* region, 10 genes were found to contain non-synonymous SNPs in coding regions (Table 2). These SNPs are listed in S9 Table.

### Acute stress responsiveness in F344 and WKY rat strains

**Fig 1:**
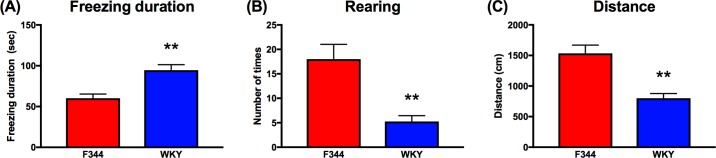
Behavioral responses to footshock of F344 and WKY adult male rats. (A) WKY adult male rats froze longer (freezing duration in seconds) following the footshocks in the CFC conditioning chamber. (B) Number of rears are significantly lower for WKYs compared to F344s. (C) F344s move around the chamber after the footshock significantly more than WKYs measured by distance traveled in cm. Data are presented as mean ± standard error of mean. *N* = 16 per group. ** *P* < 0.01 by Student’s *t*-test.

To investigate the generalizability of the findings to a different acute stress phenotype, we measured the acute stress response differences between the two strains in the conditioning part (day 1) of the CFC (Fig 1). Similar to behaviors in the DB and OFT [15, 16, 19], WKY rats exhibited significantly more freezing in response to the stress of the footshock (*t*(30) = 4.066; *P* < 0.01), which is a clear indication of a more passive stress response (Fig 1A). Furthermore, WKYs also exhibited a significantly lower frequency of rearing (*t*(29) = 4.00; *P <* 0.01), which is an avoidance response [19, 35]. WKYs also exhibited a more hypoactive response to the footshocks, measured by significantly shorter distance travelled in the chamber compared to the F344 rats (*t*(30) = 1.813; *P* < 0.01) (Fig 1B and 1C).

### Confirmation of *Stresp10* phenotypes in F344 and WKY parental strains, and in the F344.WKY*-Stresp10* congenic strain

**Fig 2:**
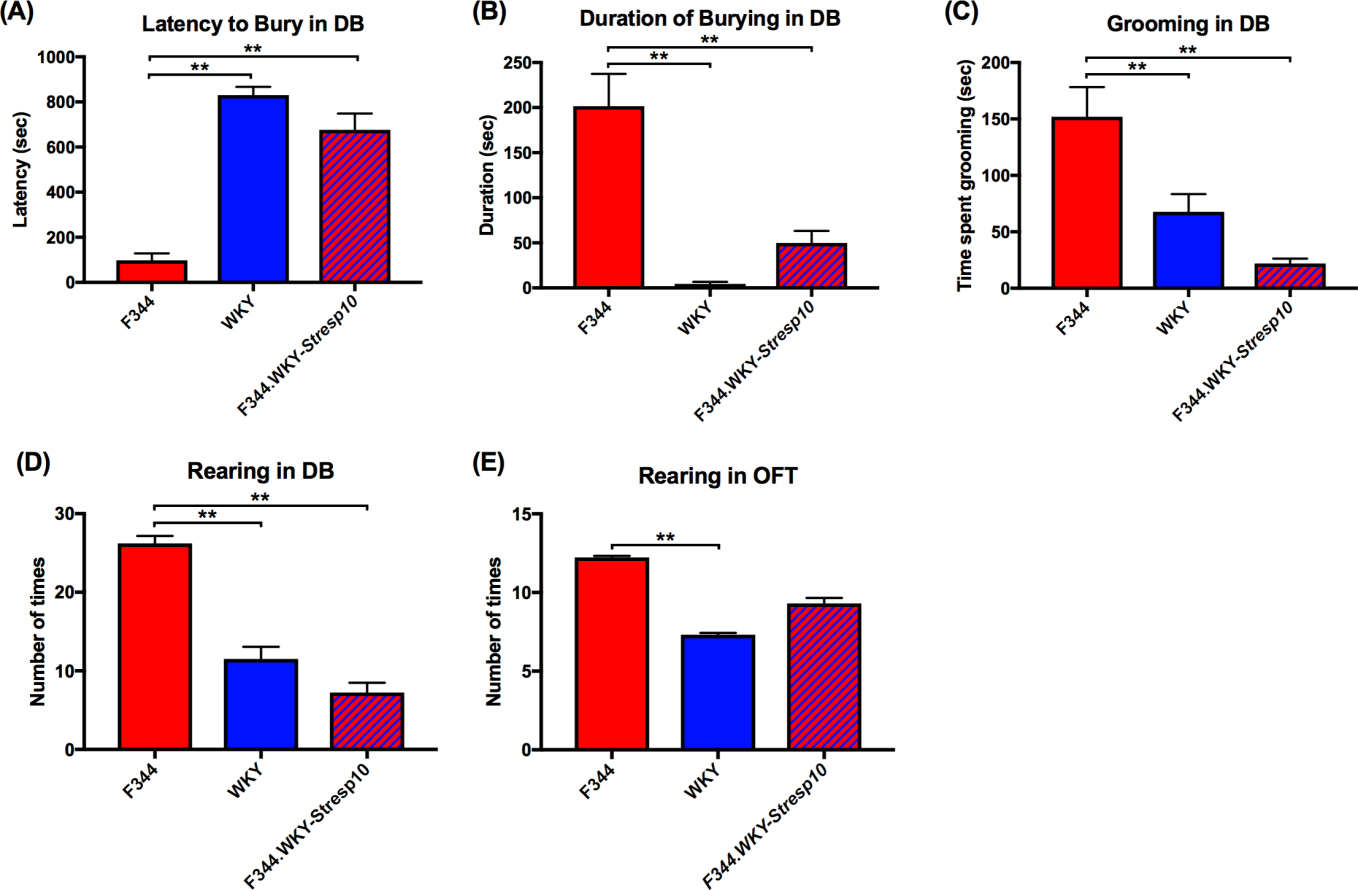
Phenotypic differences between the F344, WKY, and F344.WKY-*Stresp10* strains in the defensive burying and open field tests. (A, B) Both WKY and F344.WKY-*Stresp10* adult male rats exhibited significantly longer latency to bury and shorter duration of burying the electrified prod compared to the F344s. (C, D) Both WKY and F344.WKY-*Stresp10* rats groomed less (time spent grooming in seconds) and reared less compared to the F344s in the DB paradigm. (E) WKYs also rear significantly less than F344s in the OFT, while the number of rearing of F344.WKY-*Stresp10* did not differ significantly from either parental strain. Data are presented as mean ± standard error of mean. *N =* 13–18 per group. ** *P* < 0.01 by two-way ANOVA followed by Bonferroni’s multiple comparisons test.

The phenotypes associated with *Stesp10* include latency to bury, grooming, and rearing in the DB test, and rearing in the OFT; all of which represent an acute stress response. To confirm that the *Stresp10* QTL contributes to the variation in these behavioral acute stress responses, a congenic strain, F344.WKY*-Stresp10*, was generated (S1 Table). Fig 2 shows the phenotypic differences between the F344, WKY, and F344.WKY-*Stresp10* strains. In the DB test, both the WKY and F344.WKY-*Stresp10* rats exhibited enhanced avoidance and hypoactive responses to acute stress, measured by significantly longer latency to bury (*F*[3,46] = 44.55; *P* < 0.01) and shorter duration of burying the electrified prod (*F*[2,43] = 23.96; *P* < 0.01) compared to the F344s (Fig 2A and 2B). Furthermore, WKY and F344.WKY-*Stresp10* rats were less active showing less grooming (strain: *F*[2,43] = 11.80, *P* < 0.01) and rearing (strain: *F*[2,25] = 34.04, *P* < 0.01) compared to the F344 rats (Fig 2C and 2D). In the OFT, WKYs reared significantly less (*F*[2,87] = 14.79, *P* < 0.01) and the number of rearing for the F344.WKY-*Stresp10* were intermediary not differing from either parental strain significantly (Fig 2E).

### Quantitative expression of DEGs with sequence variations in the brain regions of F344, WKY, and F344-WKY.*Stresp10* adult male rats

Quantitative expression analyses of the 10 genes (Table 2) were carried out using RNA from the hippocampus, amygdala, and frontal cortex of F344, WKY, and congenic strain F344.WKY-*Stresp10* male rats.

**Fig 3:**
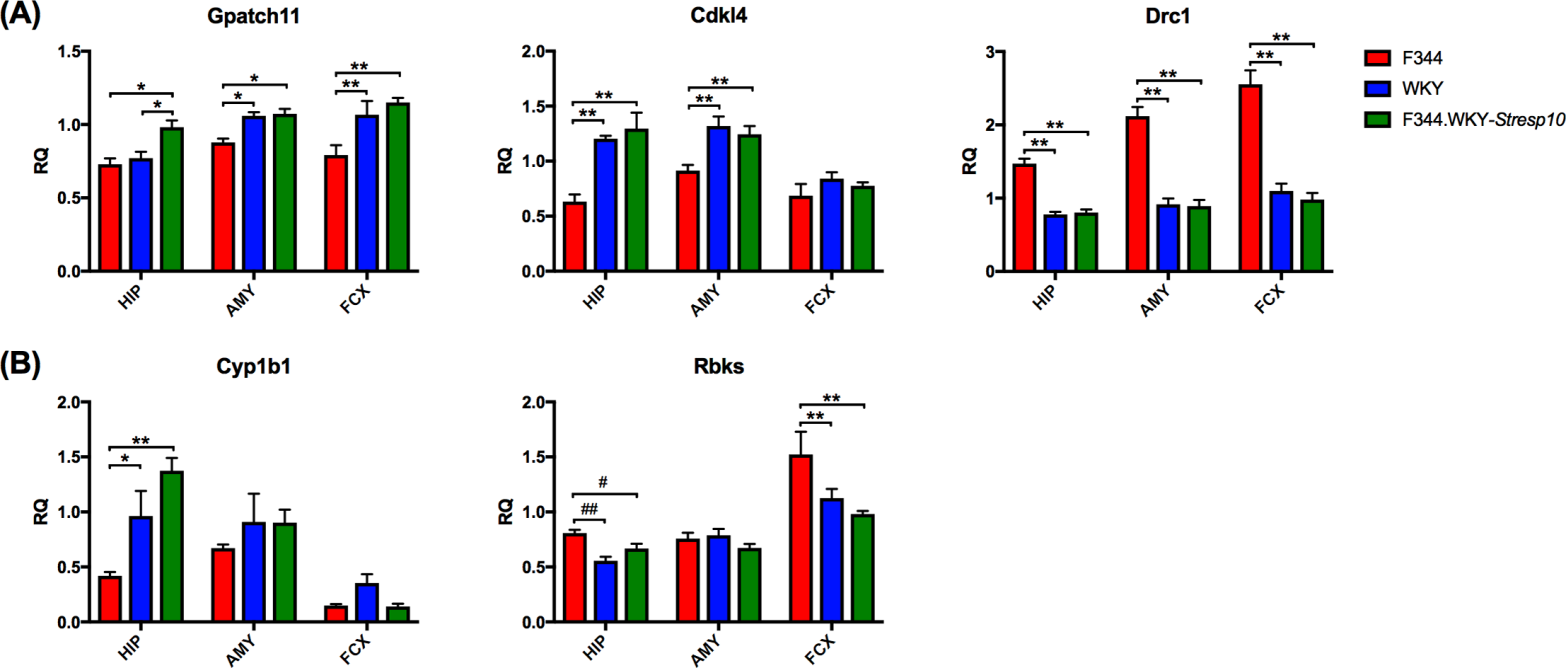
Expression analyses of candidate quantitative trait genes in the hippocampus, amygdala, and frontal cortex of F344, WKY, and F344.WKY-*Stresp10* adult male rats. (A) Candidate genes with similar expression between the WKY and F344.WKY-*Stresp10* strains, but different from the F344, in two or more brain regions. (B) Genes that express a similar pattern in only one brain region. Data are presented as mean ± standard error of mean. *N* = 5–7 per group. * *P* < 0.05 and ** *P* < 0.01 by two-way ANOVA followed by Bonferroni’s multiple comparisons test. # *P* < 0.05 and ## *P* < 0.01 by Student’s *t-*test.

Genes with similar expression between the WKY and F344.WKY-*Stresp10* strains, but different from the F344, in two or more brain regions were considered to be strong candidate QTGs that likely contribute to the variation in acute stress responsiveness between the parental strains. These candidate QTGs include *Gpatch11* (G-patch domain containing 11), *Cdkl4* (Cyclin dependent kinase like 1), and *Drc1* (Dynein regulatory complex subunit 1) (Fig 3A). Expression of *Gpatch11* differed significantly by brain region and strain (brain region: *F*[2,51] = 12.75, *P* < 0.01; strain: *F*[2,51] = 21.82, *P* < 0.01). Expression of *Drc* differed significantly by brain region and strain as well the interaction between the two (brain region: *F*[2,43] = 15.39, *P* < 0.01; strain: *F*[2,43] = 96.56, *P* < 0.01; interaction: *F*[4,43] = 3.844, *P* < 0.01). Both *Gpatch11* and *Drc1* showed significant differences in expression between the F344s and WKYs and F344s and F344.WKY-*Stresp10* rats in all three brain regions. Expression of *Cdlk4* differed significantly by brain region and strain as well as the interaction between the two (brain region: *F*[2,44] = 22.85, *P* < 0.01; strain: *F*[2,44] = 21.82, *P* < 0.01, interaction: *F*[4,44] = 3.34, *P <* 0.05). *Cdkl4* also showed the same pattern of expression in the hippocampus and amygdala, while transcript levels in the frontal cortex were relatively low.

Additionally, genes that express the same pattern but only in one brain region were considered. Sequence variations in these genes can interfere with gene expression by interacting with brain region-specific modulators, such as miRNAs, transcription factors, and binding proteins, present or absent in the specific brain region, which may or may not induce transcription [36, 37]. Only two genes, *Cyp1b1* (Cytochrome P450 family 1 subfamily B member 1) and *Rbks* (ribokinase), showed this pattern of expression. (Fig 3B). Brain region- and strain-specific expression of *Cyp1b1* showed a complex pattern (brain region: *F*[2,45] = 26.01, *P* < 0.01; strain: *F*[2,45] = 7.465, *P* < 0.01; interaction: *F*[4,45] = 3.567, *P* < 0.05). *Cyp1b1* expression was significantly greater in the WKY and the F344.WKY-*Stresp10* strains compared to F344, but only in the hippocampus. Similar to the expression of *Cyp1b1*, *Rbks* transcript levels were regulated in a brain region- and strain-specific manner (brain region: *F*[2,50] = 48.67, *P* < 0.01; strain: *F*[2,50] = 10.43, *P* < 0.01; interaction: *F*[4,50] = 4.041, *P* < 0.01). *Rbks* was expressed at significantly lower levels in the WKY and F344.WKY-*Stresp10* rats relative to the F344 rats in the frontal cortex; the hippocampal expression profile was similar but F344 and the F344.WKY-*Stresp10* showed significance only as hypothesis testing (*t*(11) = 2.57, *P <* 0.01). There were no significant differences in the expression of this gene in the amygdala. *Prkd3*, *Mta3*, *Ttc27*, *Alk*, and *Dtnb* showed no strain-specific effects.

## Discussion

The major findings of this study point to candidate QTGs that can contribute to differences in behavioral responsiveness to acute stress between the F344 and WKY strains across multiple paradigms and phenotypes. Brain region-specific gene expression differences between the F344 and WKY strains and a congenic strain, incorporating a WKY donor chromosomal regions mapped to multiple stress responsive QTLs into the F344 recipient background, identified candidate genes within this locus. Those genes that showed sequence variations between the two parental strains are proposed to be candidate QTGs. These candidate QTGs were identified using a multifaceted approach that, to our knowledge, has not been used previously. Specifically, this approach included genome-wide microarray analyses to identify DEGs within the QTL in question, followed by non-synonymous SNPs within these DEGs, the generation of the congenic strain for the behavioral consequences associated with the specific QTL, and finally the expression analysis involving all three strains with a different analytical method, the qPCR.

Human studies have identified few genetic variations associated with individual differences in behavioral responses to acute stress (for example, [38, 39]). All of these candidate genetic variations were associated with responses to fearful faces measured by multiple imaging and other methods. In contrast, animal studies have identified multiple QTL for behavioral responsiveness to acute stress, but very few QTGs have been proposed [11–14, 40, 41]. The usual methods to detect QTL, such as backcrosses, F_2_ crosses, and consomic strains [42–44], usually identify large genomic regions, with large number of genes mapped to them. Other techniques such as recombinant inbred strains, congenic strains, and heterogeneous stocks [10, 45] are able to identify smaller genomic regions, but still have no confirmed QTGs for acute stress responsiveness.

In our previous QTL studies, using the recombinant F_2_ generation of the reciprocally crossed F344 and WKY, we identified multiple QTL for behavioral responsiveness to acute stress in different paradigms. Specifically, the chromosomal region of the *Stresp10* QTL was associated with latency to bury, grooming, and rearing in the DB test, and rearing in the OFT from this cross. All of these phenotypes describe a behavioral response of the animal either to a novel environment or to an aversive shock stimulus, which are characteristically either active or passive. In the DB test, these options lead to the active behaviors of avoiding the shock by increasing the duration of burying and of rearing, or the passive response to the shock manifested by freezing and thereby increased latency to bury [46, 47]. The acute stress in the OFT is the novel environment from which the animal is trying to escape by rearing; an active response to this stress. These overlapping QTL within the *Stresp10* locus may represent pleiotropy, or multiple sequence variations interacting, resulting in a common genetic architecture underlying different behavioral responses to acute stress.

In the fear conditioning component of the CFC test, the animal can either freeze (parallel to latency to bury in the DB), explore (parallel to burying), or rear, after it receives the footshock. Exploration is an active behavior that is inversely related to freezing, while rearing is a risk-assessing behavior that encompasses exploratory, activity, and excitability components [15, 16, 19]. In both the CFC and the DB tests, the initial period of exploration allows the animal to form an associative memory between the context and the conditioning stimulus. This association has been proven to be necessary to trigger conditioned fear responses in the CFC and is essential to the learning component of the fear conditioning paradigm [48, 49]. Thus, the individual, genetic differences in these responses likely influence the degree of fear memory recall and, thereby, are of major significance. Regarding the DB test, re-exposure to the DB chamber without the shock, similar to CFC, suggested that this is also a learning paradigm [50]. Although the CFC phenotypes may not be mapped to *Stresp10*, considering the abovementioned parallels and the third rearing phenotype showing similar differences between the strains, we presumed that these acute stress phenotypes are relevant to the current study. Indeed, the patterns of candidate gene expressions reflect the behavioral measures in the CFC, OFT, and DB tests, suggesting that the candidate QTGs contribute to variations in the general behavioral response to acute stress, as we had hypothesized.

In this study, we aimed to identify candidate QTGs within the *Stresp10* QTL. We mined our previously collected genome-wide transcriptomic data to find genes in this region with brain region-specific expression that parallel the behavioral responses to acute stress in the F344 and WKY strains. The brain regions explored are the hippocampus, amygdala, and frontal cortex, which are all intimately involved in the behaviors discussed. The neural circuitry connecting the amygdala and frontal cortex are involved in the emotional responses to acute stress [51–54]. The ventral hippocampus is involved in anxiety-like responses, while the dorsal hippocampus is more involved in fear learning [55]. Because the sorting of behavioral responses to these categories is not feasible, we examined the whole hippocampus in this study. Since the goal of this study was to identify QTGs for acute stress responsiveness, the assumption was that steady-state expression of DEGs between the F344 and WKY strains will differ in all three brain regions due to sequence variations in the candidate QTGs.

As a proof-of-concept, we hypothesized that the expression of candidate QTGs in the brain of the congenic strain F344.WKY-*Stresp10* would parallel that of the WKY and differ from the F344 parental strain. This pattern would mirror the strain differences in behavioral phenotypes obtained either in the DB or the OFT. We identified sequence variations within 12 genes, of which a total of five candidate QTGs were identified. Expression of three of the genes (*Cdkl4*, *Drc1*, and *Gpatch11*) were parallel to the behavior in two or more brain regions, while the other two (*Cyp1b1* and *Rbks*) were parallel to the behavior in at least one brain region. *Cdkl4* belongs to the cyclin-dependent protein kinase family and is responsible for cell cycle progression, including transferase activity, transferring phosphorous-containing groups and protein tyrosine kinase activity [56]. *Drc1* encodes a key component of the nexin-dynein complex that regulates the assembly of ciliary dynein [57]. *Gpatch11* is involved in nucleic acid binding [58]. *Cyp1b1* encodes a member of the cytochrome *P450* superfamily of enzymes, which catalyze reactions involved in drug metabolism and synthesis of cholesterol, steroids, and other lipids [59–61]. In retinal endothelial cells, expression of *Cyp1b1* has been shown to reduce intracellular oxidative stress; although this has not been shown in neurons [62]. *Rbks* encodes a member of the carbohydrate kinase *PkfB* family and is known to catalyze the phosphorylation of ribose [63]. Although none of these genes have been directly implicated for acute stress responsiveness, their genomic location within QTLs for acute stress response suggests that they may underlie some common mechanisms of these phenotypic traits.

Interestingly, the individual candidate QTGs are link to stress-related immunoregulatory genes, including *Il5* (interleukin 5), *Btnl2* (butyrophilin-like 2), *Ifna2* (interferon alpha 2), and *Ifnl1* (interferon lambda 1) [64–67]. Both *Il5* and *Btnl2* can activate candidate QTG *Drc1*, which is known to encode a key component of the nexin-dynein complex that regulates the assembly of ciliary dynein [64, 65, 68]. While *Drc1* has never been implicated in stress responses or stress-related disorders, *Il5* has been reported to be differentially expressed in the frontal cortex of rats exposed to acute stress [69]. In a human study, elevated levels of *Il5* were associated with an increased likelihood of major depressive disorder [70, 71]. Additionally, candidate QTG *Drc1* can be activated by *Cd38* (Cd38 molecule), which encodes a multifunctional protein involved in glucose-mediated insulin secretion and immune system functioning [64, 65, 72]. In the brain, *Cd38* regulates the secretion of the neuropeptide oxytocin and is associated with several stress-related phenotypes, including social impairments in humans such as autism spectrum disorder [73]. Additionally, the *rs3796863* SNP is associated with heightened stress sensitivity and predicting social anxiety and depression in humans [73]. The other candidate QTG *Gpatch11* can also be activated by *Ifna2* and *Ifnl1*, which are both interferon immunosuppressor genes. Interestingly, *Ifna2* is a pleiotropic cytokine that triggers immune responses, hypothalamic-pituitary-adrenal axis abnormalities and disturbances in brain metabolism resembling those in depressive states [74]. *Ifna2* is also known to induce memory, concentration, and mood disturbances when administered as a therapeutic [74]. In differentiating neurons, the expression of *Ifna2* affects their response to inflammatory cytokines, which is consistent with molecular mechanisms involved in schizophrenia and autism spectrum disorder [75].

One inherent limitation of this study stems from the heterozygous region of the congenic strain, which we excluded from our analyses. Other candidate QTGs could be mapped to this region. Among the other limitations of this study is that it focuses on candidate QTGs with non-synonymous sequence variations in their coding regions. It is known that the large majority of sequence variations are in non-coding regions that may act as cis-regulatory and/or trans-acting modules. This makes identification of candidate QTGs for behavioral and psychiatric phenotypes more difficult [76]. However, our presumption that the SNPs within the QTGs affect the expression of these genes prompted us to first investigate coding region non-synonymous sequence variations. Furthermore, among inherited gene variations in humans, the non-synonymous SNPs in coding regions that lead to changes in amino acid in protein expression are most relevant to human inherited diseases [32]. Additionally, these candidate QTGs with cis-regulated expression changes can affect gene expression in trans, as described in Bryois et al. [77]. Future studies will examine sequence variations between F344 and WKY within the QTL in non-coding sequences conserved across species, as described in Yoshihara et al. [78]. We will also focus on sequence variations in microRNAs with known gene targets (www.targetscan.org and www.exiqon.com/microrna-target-prediction).

Taken together, our findings indicate that strain differences in acute stress responsiveness are generalizable across multiple behavioral paradigms. The unique approach of transcriptomics combined with sequence variations within a specific QTL in the different parental and congenic strains identified unique QTGs that might contribute to variations in the behavioral responses to acute stress. The role of these candidate genes in the behavioral response to stress should be confirmed in future studies requiring brain region- and strain-specific transgenic approaches.

## Supporting information

S1 Table Genotype of the *Stresp10* region of the F344.WKY-*Stresp10* congenic strain. (XLSX)

S2 Table List of primer sequences for RT-PCR analysis. (XLSX)

S3 Table List of DEGs between F344 and WKY strains in the hippocampus. (XLSX)

S4 Table List of DEGs between F344 and WKY strains in the amygdala. (XLSX)

S5 Table List of DEGs between F344 and WKY strains in the frontal cortex. (XLSX)

S6 Table List of DEGs mapped within *Stresp10* in the hippocampus. (XLSX)

S7 Table List of DEGs mapped within *Stresp10* in the amygdala. (XLSX)

S8 Table List of DEGs mapped within *Stresp10* in the frontal cortex. (XLSX)

S9 Table List of SNPs found within the coding regions of DEGs mapped within *Stresp10*. (XLSX)
